# Integration of soil microbiology and metabolomics to elucidate the mechanism of the accelerated infestation of tobacco by the root-knot nematode

**DOI:** 10.3389/fmicb.2024.1455880

**Published:** 2024-08-23

**Authors:** Yinghua Sang, Ke Ren, Yi Chen, Bin Wang, Yufang Meng, Wenbing Zhou, Yonglei Jiang, Junju Xu

**Affiliations:** ^1^College of Tobacco Science, Yunnan Agricultural University, Kunming, China; ^2^Yunnan Academy of Tobacco Agricultural Sciences, Yuxi, China; ^3^Yuxi Branch of Yunnan Provincial Tobacco Company, Yuxi, Yunnan, China

**Keywords:** tobacco root-knot nematode, tobacco, rhizosphere soil, microbiology, metabolomics

## Abstract

**Introduction:**

Tobacco root-knot nematode (TRKN) disease is a soil-borne disease that presents a major hazard to the cultivation of tobacco, causing significant reduction in crop quality and yield, and affecting soil microbial diversity and metabolites. However, differences in rhizosphere soil microbial communities and metabolites between healthy tobacco soils and tobacco soils with varying degrees of TRKN infection remain unclear.

**Methods:**

In this study, diseased rhizosphere soils of tobacco infected with different degrees of TRKN [severally diseased (DH) soils, moderately diseased (DM) soils, and mildly diseased (DL) soils] and healthy (H) rhizosphere soils were collected. Here, we combined microbiology with metabolomics to investigate changes in rhizosphere microbial communities and metabolism in healthy and TRKN-infected tobacco using high-throughput sequencing and LC-MS/MS platforms.

**Results:**

The results showed that the Chao1 and Shannon indices of bacterial communities in moderately and mildly diseased soils were significantly higher than healthy soils. The Proteobacteria, Actinobacteria, Ascomycota, Burkholderia, *Bradyrhizobium* and *Dyella* were enriched in the rhizosphere soil of healthy tobacco. Basidiomycota, Agaricales, Pseudeurotiaceae and *Ralstonia* were enriched in severally diseased soils. Besides, healthy soils exhibited a relatively complex and interconnected network of bacterial molecular ecologies, while in severally and moderately diseased soils the fungal molecular networks are relatively complex. Redundancy analysis showed that total nitrogen, nitrate nitrogen, available phosphorus, significantly affected the changes in microbial communities. In addition, metabolomics results indicated that rhizosphere soil metabolites were significantly altered after tobacco plants were infected with TRKNs. The relative abundance of organic acids was higher in severally diseased soils. Spearman’s analyses showed that oleic acid, C16 sphinganine, 16-hydroxyhexadecanoic acid, D-erythro-3-methylmalate were positively correlated with Basidiomycota, Agaricales, *Ralstonia*.

**Discussion:**

In conclusion, this study revealed the relationship between different levels of TRKN invasion of tobacco root systems with bacteria, fungi, metabolites and soil environmental factors, and provides a theoretical basis for the biological control of TRKN disease.

## 1 Introduction

Root-knot nematode disease (RKN) is one of the major soil-borne diseases caused by plant-parasitic root-knot nematodes (*Meloidogyne* spp.) invading plant roots. It has become the second major category of plant diseases in agricultural production, following fungal diseases (Chalivendra, 2021). Tobacco is one of the major economic crops in China and can be susceptible to RKNs, which severely affecting yield and quality of tobacco leaf (Barker and Weeks, 1991). TRKNs disease has been discovered in major tobacco growing regions in China, with yield losses ranging from 30 to 50%, and the severity of the damage increases every year (Tong et al., 2022). Using chemical insecticides is the most effective method of controlling RKNs. However, due to the long-term misuse of such pesticides, root-knot nematodes have developed strong resistance, while simultaneously posing a high toxicity risk to humans, animals, and other beneficial microorganisms in the soil, causing environmental pollution and related issues. Therefore, this approach has limited efficacy in terms of control (Huang et al., 2016; Zhang et al., 2018; Hu et al., 2023). Biological control primarily uses microbial agents, plant-derived insecticides, and endogenous plant hormones to eliminate nematodes. Due to its non-polluting, environmentally friendly, and long-lasting advantages, biological control has emerged as a vital component of integrated pest management systems for tobacco diseases and pests. Research has showed that the use of biocontrol bacteria can achieve good results in the control of plant diseases (Penton et al., 2014; Cha et al., 2016; Liang et al., 2019; Pires et al., 2022).

Soil microorganisms are a major component of soil ecosystems and directly participate in organic matter decomposition and nutrient cycling processes, they are closely related to plant growth. Clarifying the correlation between soil microbes and the balance of soil ecosystems is necessary for disease prevention and crop health (Chen et al., 2019). Studies have showed that the high incidence of soil-borne diseases may be due to the deterioration of the soil microbial environment (Wei et al., 2015; Gao et al., 2020). Studies have revealed significant differences between soils affected by healthy and soil-borne diseased soils regarding microbial composition, in banana non-diseased soils, beneficial microorganisms such as Bacillus, Streptomyces, and Pseudomonas were more abundant than soils infected with wilt disease (*Musa* spp.) (Zhou et al., 2019). The abundance of Pseudomonas in healthy soils are significantly higher than in soils where tobacco black shank disease occurs, the addition of *Bacillus subtilis* S719 is beneficial in reducing the incidence of tobacco black shank disease (Ma et al., 2023). Therefore, a theoretical basis for the prevention and control of soil-borne diseases can be provided by studying rhizosphere soil microecosystem in tobacco roots with different levels of TRKN infection (Wang et al., 2022; Zhao et al., 2023).

The development of high-throughput sequencing technology provides a way to study the relationship between the microbial community of the plant rhizosphere microbial community and root-knot nematode disease. High-throughput sequencing technologies have been used to explore the microbial composition and diversity of tobacco roots (rhizosphere and endophytic) at different growth stages in typical tobacco RKN-infected areas for 2 consecutive years, the results were observed that root-knot nematode infection altered the α-diversity and microbial composition of the root-associated microbiota (Cao et al., 2022). Analysis of bacterial communities in tobacco plants infected with RKNs and in healthy plants demonstrated differences in community structure and metabolic function between diseased and healthy rhizosphere soils (Tong et al., 2022). In healthy soils, there was a negative correlation between Pseudomonas and the appearance of root-knot nematode disease, while Bryobacter, Variibacter, Coniochaeta, and Metarhizium were positively associated with disease occurrence in infected soils (Huang et al., 2020). Studies have revealed significant differences in the rhizosphere bacterial microbiota and diversity among different levels of TRKNs infection (Lu et al., 2023). At the same time, plant and rhizosphere microbial metabolites are important in regulating plant health (Bi et al., 2021). Results from previous studies have showed that metabolites (ribose, lactic acid, xylose, mannose, maltose, gluconolactone and ribitol) from the rhizosphere of tomato (*Solanum lycopersicum*) plants suffering from wilt disease (caused by *Ralstonia solanacearum*) are effective in protecting tomato, pepper (*Capsicum annuum*) and eggplant (*Solanum melongena*) from invasion by the pathogen (Wen et al., 2023). Metabolomics is the emerging discipline of simultaneous qualitative and quantitative analysis of all low molecular weight (<1,000 Da) metabolites of an organism or cell during a specific physiological period. At present, metabolomics technology has become one of the most important approaches to further investigate the functional mechanisms of soil disease suppression (Ros et al., 2020). Despite numerous studies indicating the inseparable relationship between changes in the structure and diversity of the rhizosphere soil microbial community and the occurrence of RKN disease, there is limited research on microbial communities and metabolites in tobacco rhizosphere soil under varying degrees of infection.

In this research, high-throughput sequencing and non-targeted metabolomics techniques were combined to analyze the variance in diversity, community composition, molecular networks and metabolites of rhizosphere soil microbiota between healthy and tobacco with varying degrees of RKNs infection under natural growth conditions. Our research aims: (1) to explore the impact of varying degrees of RKN infestation on soil chemical properties, microbiota and metabolites within the tobacco rhizosphere, and (2) to elucidate the microbial mechanisms underlying the increasing severity of RKN disease.

## 2 Materials and methods

### 2.1 Sample collection

In a tobacco field located in Guangnan County, Wenshan Prefecture, Yunnan Province (longitude: E104°34′45″, latitude: N23°57′52″, elevation: 1,412 m), the same tobacco variety (Yunyan 116) was planted and managed under conventional cultivation practices without any treatment against root-knot nematodes throughout the growth period. Tobacco is a major economic source, while the incidence of tobacco root-knot nematode disease is severe, causing huge economic losses.

After the maturity of the tobacco leaves and the completion of harvesting, samples were collected, classified by the presence or absence of root nodules on the tobacco roots. Soil samples were labeled diseased (D) soil when plant roots exhibited root-knot nematode infestation and healthy (H)soil when no root nodules were present. Based on the number of nodules and their decay status on the roots, the D was divided into three levels: severally diseased (DH) soils, moderately diseased (DM) soils, and mildly diseased (DL) soils. The rhizosphere soil from the root system of tobacco plants that includes primary and secondary roots severally infested with root-knot nematodes was designated as DH, representing the rhizosphere soil of tobacco plants that were severally infected with root-knot nematodes. The rhizosphere soil from the root system of tobacco plants that had one-third to one-half of the roots had nodules was labeled DM, representing rhizosphere soil in tobacco plants that were moderately infected with root-knot nematodes. The rhizosphere soil from the root system of tobacco plants that had less than one-fourth of the roots had a few nodules was labeled DL, representing rhizosphere soil in tobacco plants that were mildly infected with root-knot nematodes (Tong et al., 2022; Lu et al., 2023). Careful excavation of the tobacco roots was performed, shaking off non-rhizosphere soil. Twenty-four tobacco plants were acquired for the study, with six plants each selected from the same disease level. The rhizosphere soil (0-5 mm from the roots) was gently collected to obtain four rhizosphere soil samples (Peiffer et al., 2013). The samples were classified into two parts: one set for the assessment of soil physicochemical properties and the other set stored at a temperature of −80°C for subsequent analysis of soil microbiota.

### 2.2 Determination of soil chemical properties

The following soil chemical properties were measured in accordance to the techniques delineated in the reference by Lu (2000). each treatment had three replications. After air-drying the soil samples: Soil pH was determined in 1:5 ratios (weight: volume) of soil with distilled water using a pH meter (PHS-3\u00B0C, Shanghai). The semi-micro Kjeldahl digestion method was used to determine the total nitrogen (TN) content in the soil. The H_2_SO_4_-HCLO_4_ digestion was applied and the molybdenum-antimony anti-colorimetric technique was used to measure total phosphorus (TP) in the soil. The NaOH fusion flame photometry technique was applied for the measurement of total potassium (TK) in the soil. The available nitrogen (AN) content of the soil was measured by means of the alkaline hydrolysis diffusion technique. The sodium bicarbonate extraction-molybdenum-antimony resistance colorimetric technique was applied to extract and measure the soil available phosphorus (AP). Soil available potassium (AK) was measured by extraction with 1.0 M ammonium acetate for 30 min. It was measured by flame photometry. Soil nitrate nitrogen (NO_3_^−^-N) and ammonium nitrogen (NH_4_^+^-N) were determined using the continuous flow analyzer method.

### 2.3 DNA extraction and sequencing

DNA was extracted from 0.5 g of soil samples using the TGuide S96 Magnetic Bead Soil/Feces Genomic DNA Extraction Kit (Tian gen Biotech Co., Ltd., Beijing, China, Model: DP812) following the given protocol. The V3-V4 region of the bacterial 16S rRNA gene was amplified using primers 338F (5’-ACTCCTACGGGAGGC AGCA-3′) and 806R (5’-GGACTACHVGGGTWTCTAAT-3′), while the ITS1 region of fungal ITS was amplified using primers ITS1F (5’-CTTGGTCATTTAGAGGAAGTAA-3′) and ITS2 (5’-GCTG CGTTCTTCATCGATGC-3′). The PCR amplification reaction system contained 25 ng of DNA, 0.3 μ L of Vn F (10 μ M), 0.3 μL of Vn R (10 μM), 5 μ L of OD FX Neo Buffer, 2 μ L of NTP (2 mM each), 0.2 μ L of KOD FX Neo, and ddH_2_O to adjust the total volume to 10 μ L. The PCR amplification program is set as follows: 98°C for 30 s; 95°C for 3 s, 50°C for 30 s, 72°C for 40 cycles, 72°C for 7 min, and storage at 4°C. The PCR products were homogenously combined at equimolar concentrations and subsequently subjected to high-throughput sequencing on the Illumina Novaseq 6,000 platform, and FASTQ format raw data were obtained. The raw sequence data reported in this paper have been deposited in the Genome Sequence Archive in National Genomics Data Center, China National Center for Bioinformation / Beijing Institute of Genomics, Chinese Academy of Sciences (under accession project numbers CRA012239 and CRA012301 for 16S rRNA and ITS rRNA genes, respectively), that are publicly accessible at https://ngdc.cncb.ac.cn/gsa.

### 2.4 Metabolite extraction and LC–MS/MS analysis

The 100 mg soil samples were individually grounded with liquid nitrogen and the homogenate was resuspended with 500 μL prechilled 80% methanol by well vortex. The samples were incubated on ice for 5 min and then were centrifuged at 15,000 g, 4°C for 20 min. Some of supernatant was diluted to final concentration containing 53% methanol by LC–MS grade water. The samples were subsequently transferred to a fresh Eppendorf tube and then were centrifuged at 15,000 g, 4°C for 20 min. Finally, the supernatant was injected into the LC–MS/MS system analysis.

LC–MS/MS analyses were performed using a Vanquish UHPLC system (ThermoFisher, Germany) coupled with an Orbitrap Q Exactive^™^ HF-X mass spectrometer (Thermo Fisher, Germany) in Gene Denovo Co., Ltd. (Guangzhou, China). Samples were injected onto a Hypesil Gold column (100 × 2.1 mm, 1.9 μm) using a 17-min linear gradient at a flow rate of 0.2 mL/min. The eluents for the positive polarity mode were eluent A (0.1% FA in Water) and eluent B (Methanol). The eluents for the negative polarity mode were eluent A (5 mM ammonium acetate, pH 9.0) and eluent B (Methanol). The solvent gradient was set as follows: 2% B, 1.5 min; 2–100% B, 12.0 min; 100% B, 14.0 min; 100–2% B, 14.1 min; 2% B, 17 min. Q Exactive^™^ HF-X mass spectrometer was operated in positive/negative polarity mode with spray voltage of 3.2 kV, capillary temperature of 320°C, sheath gas flow rate of 40 arb and aux gas flow rate of 10 arb.

### 2.5 Bioinformatic analysis

The raw data was first subjected to quality filtering using Trimmomatic (version 0.33) (Bolger et al., 2014). Next, Cutadapt (version 1.9.1) was utilized to select and eliminate primer sequences (Mohideen et al., 2020). Subsequently, USEARCH (version 10) was used to assemble the paired-end reads (Edgar, 2013) and UCHIME 8.1 (Edgar et al., 2011) was employed to remove chimeras. Ultimately, high-quality sequences were obtained and used for subsequent analysis.

### 2.6 Statistical analysis

QIIME2 (version 2020.6) was used to calculate the Alpha diversity (Chao1 and Shannon indexes), perform the principal coordinate analysis (PCoA) based on Bray–Curtis dissimilarity, and conduct Beta diversity analysis using UPGMA. Linear discriminant analysis effect size (LEfSe) was employed to identify statistically different biomarkers at different taxonomic levels. The analysis was performed using BMKCloud.^1^ Using the molecular ecological network analyses pipeline (MENAP)^2^ of the random matrix theory (RMT) constructs the co-occurrence network of soil microbial communities under tobacco planting under different treatments (Deng et al., 2012). The network graph was plotted using Gephi 0.10.1. Functional diversity of bacterial and fungal communities was predicted using Tax4Fun and FUNGuild, respectively. Redundancy analysis (RDA) and Pearson correlation analysis were conducted to assess the relationship between the soil microbiota and environmental factors. The figure was plotted using BMKCloud (see footnote 1). Use supervised orthogonal partial least squares analysis (OPLS-DA) (VIP > 1, *p* < 0.05) to distinguish the overall difference in metabolic profile between groups and find the difference metabolites between groups. The differentially expressed metabolites were mapped to the KEGG Pathway database.

The entirety of the collected data was stored within Microsoft Excel 2007. One-way analysis of variance (ANOVA) followed by Tukey’s post-hoc test to examine significant differences data of different treatments. All statistical analyses were performed using IBM-SPSS software (version 24.0; SPSS Inc. Chicago, IL, United States). Ultimately, data visualization was carried out using Origin 2021 software.

## 3 Results

### 3.1 Soil physicochemical properties in healthy and TRKN soils

The contents of total potassium (TK), available phosphorus (AP), and ammonium nitrogen (NH_4_^+^-N) were significantly higher in healthy soils than diseased soils, while total nitrogen (TN), available potassium (AK), hydrolytic nitrogen (AN), organic matter (OM), and pH were significantly lower (Table 1). Total phosphorus (TP) was significantly higher in healthy soils compared to (DM) and (DH) soils, while nitrate nitrogen (NO_3_^−^-N) was significantly lower. Severally diseased soils had considerably elevated AK, hydrolytic nitrogen (AN), and nitrate nitrogen (NO_3_^−^-N) levels than (DM) and (DL) soils (Table 1). (DM) and (DH) soils showed no significant differences in total nitrogen (TN) and organic matter (OM), but all these were significantly higher than (DL) soils (Table 1).

**Table 1:** Chemical properties of tobacco plant rhizosphere soils at different levels of disease severity.

Treatment	TN (g kg^−1^)	TP (g kg^−1^)	TK (g kg^−1^)	AK (mg kg^−1^)	AN (mg kg^−1^)	AP (mg kg^−1^)	OM (g kg^−1^)	NH_4_^+^-N (mg kg^−1^)	NO_3_^−^-N (mg kg^−1^)	pH
H	0.46c	0.37a	8.37a	632.77c	27.30c	83.99a	11.56c	84.46a	0.61c	5.90b
DL	0.54b	0.36a	7.48b	579.27d	34.07b	80.11b	18.51b	55.40b	1.26c	7.15a
DM	0.78a	0.31b	7.82b	689.07b	33.48b	65.10c	21.82a	51.60c	6.62b	7.12a
DH	0.82a	0.32b	7.72b	846.53a	37.62a	65.35c	21.08a	48.45d	7.44a	7.14a

DH represents severally diseased soil; DM represents moderately diseased soil; DL represents mildly diseased; H represents healthy soil. According to Duncan’s post hoc test, different small letters within the same columns indicate significant differences (*p* < 0.05).

### 3.2 Soil microbial community composition in healthy and TRKN soils

**Figure 1:**
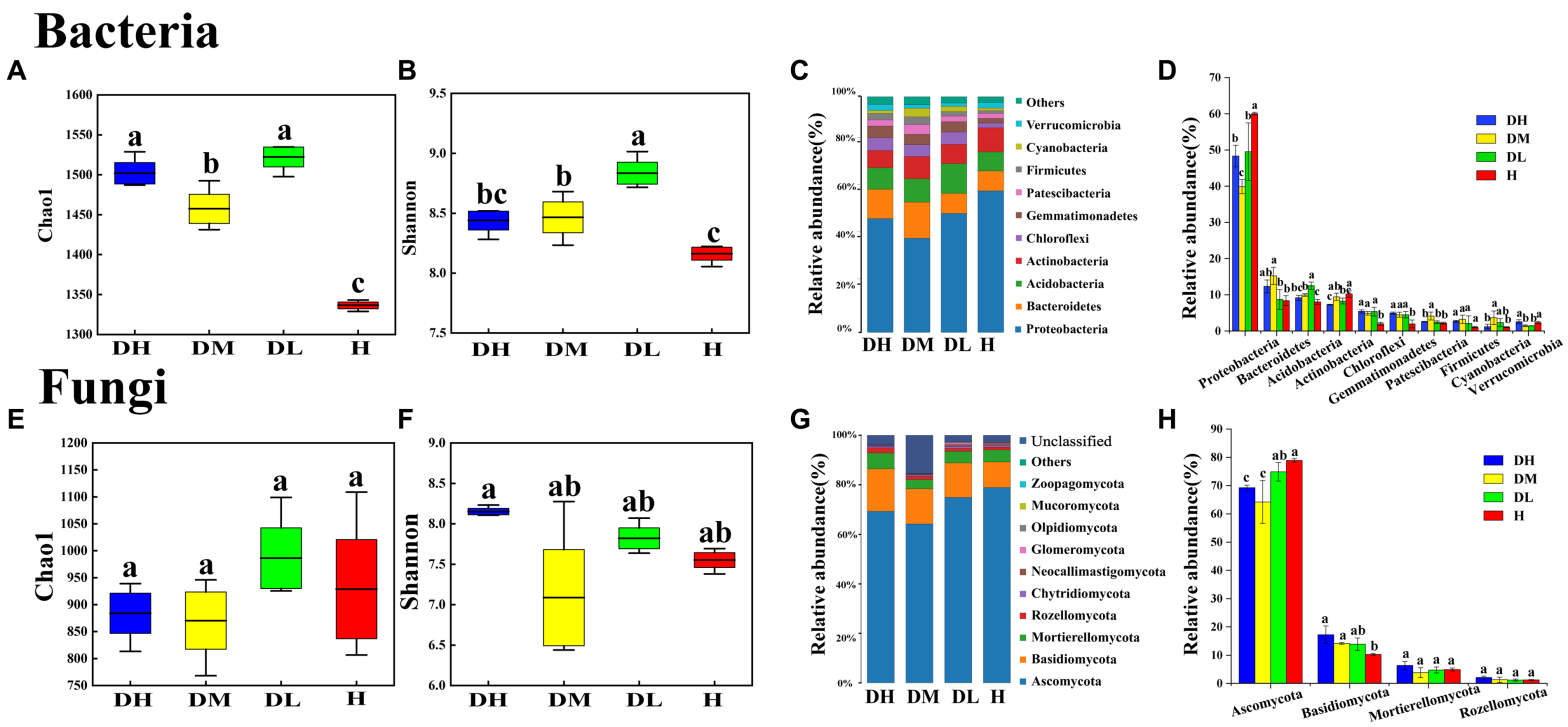
Box plots show the variation of Chao1 and Shannon indices and a histogram (relative abundance) of the composition phylum of bacterial community in healthy and diseased soils. Chao1 and Shannon indices of bacterial **(A,B)** and fungal **(E,F)** communities; community composition at bacterial **(C)** and fungal **(G)** phylum levels; histogram of relative abundance differences at bacterial **(D)** and fungal **(H)** phylum levels. Different letters within the graph indicate significant differences between the samples, subjected to one-way analysis of variance (ANOVA). For discerning treatment disparities, Duncan’s test was used, with significance set at *p* < 0.05. DH represents severally diseased soil; DM represents moderately diseased soil; DL represents mildly diseased soil; H represents healthy soil.

After quality control and filtering of the sequencing data, 945,398 bacterial clean reads and 1,594 bacterial OTUs were obtained, and an average of 78,783 clean reads produced per sample. In addition, 955,825 fungal clean reads and 1884 fungal OTUs were obtained, and an average of 79,652 clean reads produced per sample. The bacterial Chao1 and Shannon indexes of DM and DL soils were significantly higher than H soil (Figures 1A,B). In the diseased soils, the bacterial Shannon index of DL soils was significantly higher than DM and DH soils (Figure 1B). However, there were no significant difference in fungal Chao1 and Shannon indices between healthy and diseased soils (Figures 1E,F).

The bacterial and fungal populations within diseased (DH, DM, DL) soils were significantly separated from those of healthy (H) soils, indicating substantial alterations in the microbial communities of soils with tobacco root-knot nematode infection (Supplementary Figure S1). The rhizosphere bacterial community was primarily composed of phyla Proteobacteria (39.89% ~ 60.08%), Bacteroidetes (8.39% ~ 15.25%), Acidobacteria (8.08% ~ 12.53%), Actinobacteria (7.31% ~ 10.25%), Chloroflexi (2.00% ~ 5.53%), and Gemmatimonadetes (1.97% ~ 4.95%), which together accounted for up to 90% of the total bacterial abundance (Figure 1C). The abundance of Proteobacteria and Actinobacteria from H soils were significantly higher than diseased soils, while Chloroflexi and Gemmatimonadetes were significantly lower (Figure 1D). The abundance of Acidobacteria in DL soils was significantly higher than DH and DM soils. Actinobacteria of DH soils was significantly lower than DM and DL soils (Figure 1D). The rhizosphere fungal community is primarily composed of Ascomycota (64.20% ~ 78.89%), Basidiomycota (10.30% ~ 17.29%), Mortierellomycota (3.82% ~ 6.35%), and Rozellomycota (1.18% ~ 2.10%), which account for up to 90% of the total fungal count (Figure 1G). The abundance of Ascomycota in DH and DM soils were significantly lower than in DL and H soils, while Basidiomycota were significantly higher than in H soils (Figure 1F).

According to the LEfSe analysis, it had 27 bacteria clades presented statistically significant differences (Supplementary Figures S2A,B). Proteobacteria (Phylum), Actinobacteria (Phylum), Gammaproteobacteria (class), Acidobacteriia (class), Xanthomonadales (order), Burkholderia (family), Bradyrhizobium (genus) and Dyella (genus) were enriched mainly in H soils. Acidobacteria (Phylum) was enriched in DL soils. Patescibacteria (Phylum) and Bacteroidetes (Phylum) were enriched in DM soils. Betaproteobacteriales (order) was enriched in DH soils. In fungal community, Ascomycota (Phylum) and Venturiale (order) were enriched mainly in H soils, whereas Neocallimastigomycota (Phylum) was more enriched in the DL soils. Basidiomycota (Phylum), Agaricales (order), Holtermanniales (order) were located mainly in the DH soils (Supplementary Figures S2C,D).

### 3.3 Co-occurrence networks of microbial communities

**Figure 2:**
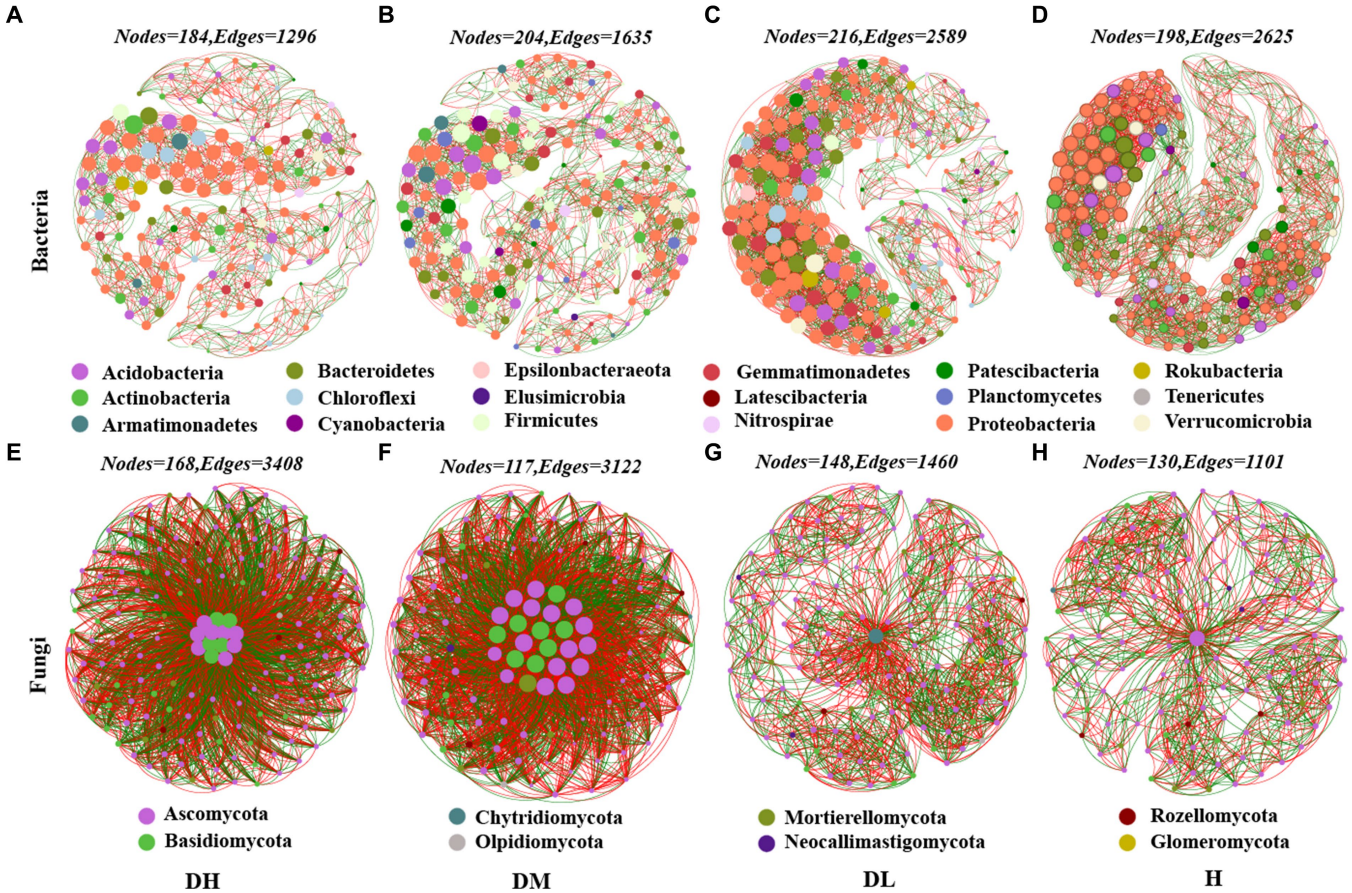
Molecular ecology networks (MENs) based on OTUs. **(A)** DH Soil Bacteria Network. **(B)** DM Soil Bacteria Network. **(C)** DL Soil Bacteria Network. **(D)** H Soil Bacteria Network. **(E)** DH Soil Fungus Network. **(F)** DM Soil Fungus Network. **(G)** DL Soil Fungus Network. **(H)** H Soil Fungus Network. Nodes refer to microbial OTU, while links refer to the relationship between nodes. The red line represents a positive correlation, and the green links represent a negative correlation. The same color represents the same phyla.

The rhizosphere bacterial microbial network had 198 nodes and 2,625 edges in the healthy soil, but the nodes and edges of diseased soils ranged from 184 to 216 and 1,296 to 2,589, respectively (Figures 2A–D). The average *K* of diseased soils ranged from 14.09 to 23.97 was lower than healthy soil (26.52) (Supplementary Table S1). However, the average *K* and edges were the highest in the network of DL soil (23.97), followed by DM and DH soils. The average path distance of H soils was 4.68 that lower than diseased soil ranged from 6.174 to 7.08 (Supplementary Table S1). The average path distance was the highest in the network of DH soils, followed by DM soils and DL soils. In the healthy soils network, the top five nodes with the highest connectivity at the phylum level were Bacteroidetes, Proteobacteria and Actinobacteria (Supplementary Table S2). In the fungal molecular network, the number of edges, average degree, and graph density of H soils were smaller than those of diseased soil, while the average path length followed the order of DM < DH < H < DL. DM and DH soils had the highest average degree, graph density, and shortest average path length (Figures 2E–H, Supplementary Table S3). Furthermore, among fungi with connectivity greater than 100, the Basidiomycota accounted for 38.46 and 34.78% in DH and DM, respectively, while it was absent in DL and H (Supplementary Table S4). It is obvious that the bacterial network becomes looser as the severity of disease increases, while the fungal network becomes more complex.

### 3.4 The prediction of microbial function

**Figure 3:**
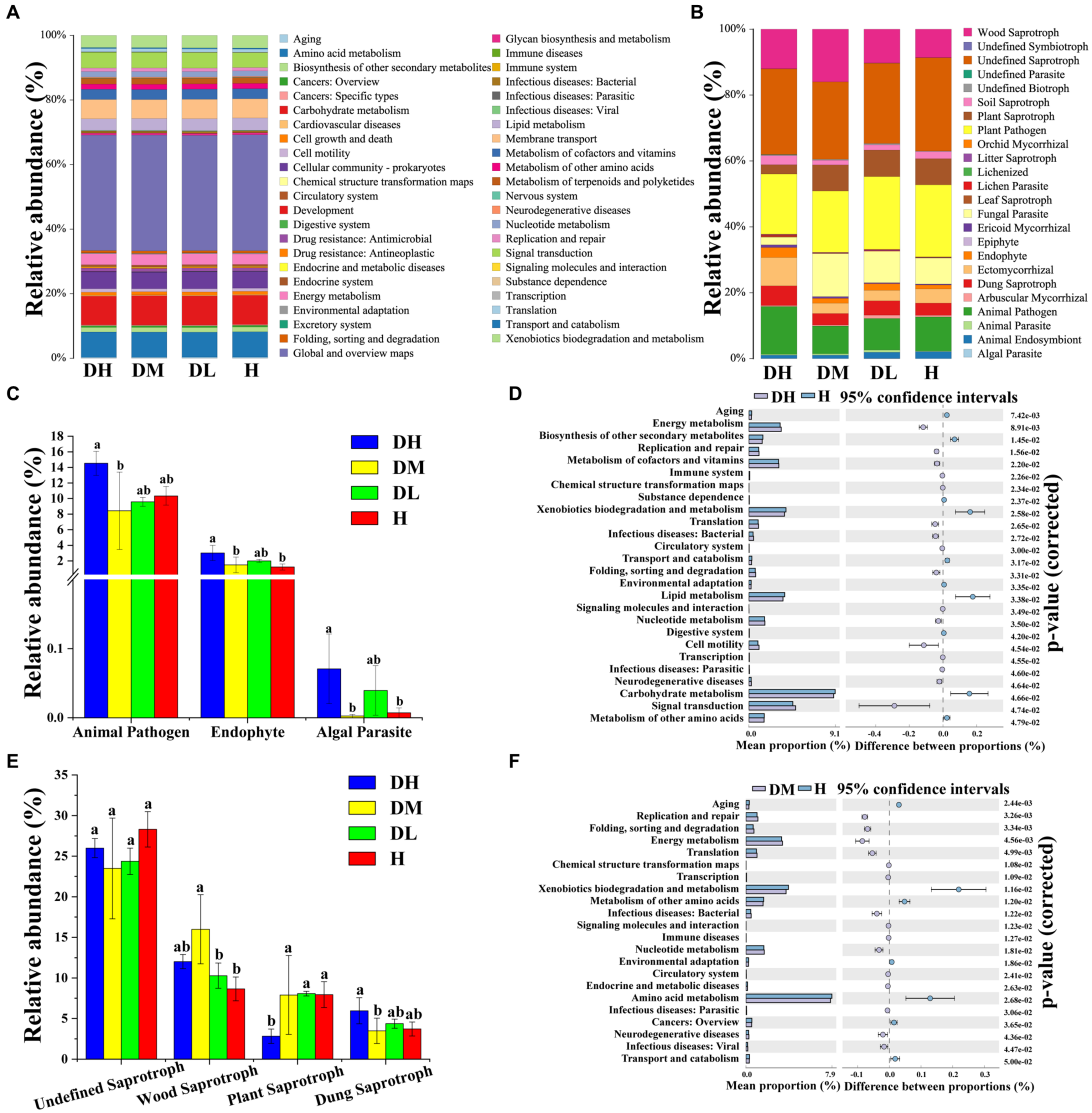
Predicted functions of the bacterial and fungal communities. Histograms of bacterial functional prediction **(A)**; histograms of fungal functional prediction **(B)**; differential pathway maps between DH and H **(B)**; histogram of differences in the abundance of nutritional types of fungal pathotypes. **(C)**; differential pathway maps between DH and H **(D)**; histogram of differences in the abundance of saprophytic nutritional types among fungi **(E)**; differential pathway maps between DM and H **(F)**. The graphs **(A,D,F)** represent the differential analysis of KEGG metabolic pathways at the second hierarchical level. Different colors denote different samples or groups. The left side represents the proportion of functional abundance in the two sample groups, the middle shows the proportion of differences in functional abundance within a 95% confidence interval, and the rightmost value is the *p*-value. Various letters represent statistically significant differences (*p* < 0.05) between treatments.

The abundance of environmental and genetic information processing, and human diseases was significantly higher in DH soils compared to H soils, but the abundances of metabolism and organismal systems were significantly (Supplementary Table S5). A total of 45 pathways were involved in the four treatments. The outcomes highlighted 26 and 22 significantly different pathways between DH and H and DM and H, respectively. Compared to H soils, the abundance of energy metabolism enhanced substantially in DH and DM soils, while the abundance of aging decreased significantly (Figures 3A,D,F). This indicates significant differences in rhizosphere soil bacterial functional profiles between TRKN infected plants and healthy plants. Furthermore, the impact on bacterial functions becomes more pronounced as the severity of disease increases.

The fungal community can be divided into 24 nutritional mode guilds. The top five fungal nutritional modes were undefined saprotroph, plant pathogen, wood saprotroph, animal pathogen, and fungal parasite (Figure 3B). The abundances of animal pathogens, endophytes, and algal parasites in DH soils were significantly higher than in DM, and endophytes and algal parasites were significantly higher than in H soils (Figure 3C). The abundance of the undefined saprotroph, which had the highest abundance, did not showed a significant difference among varying treatments but exhibited a decreasing trend in infected soil compared to H soil. The abundance of wood saprotroph in DM soils was the highest and significantly higher than in DL and H soils, followed by DH soils. The abundance of plant saprotroph in DH soils was the lowest and significantly lower than in the other three treatments. The abundance of dung saprotroph in DH soils was the highest and significantly higher than DM soils (Figure 3E).

### 3.5 Relationship between soil physicochemical properties and microbial structure

**Figure 4:**
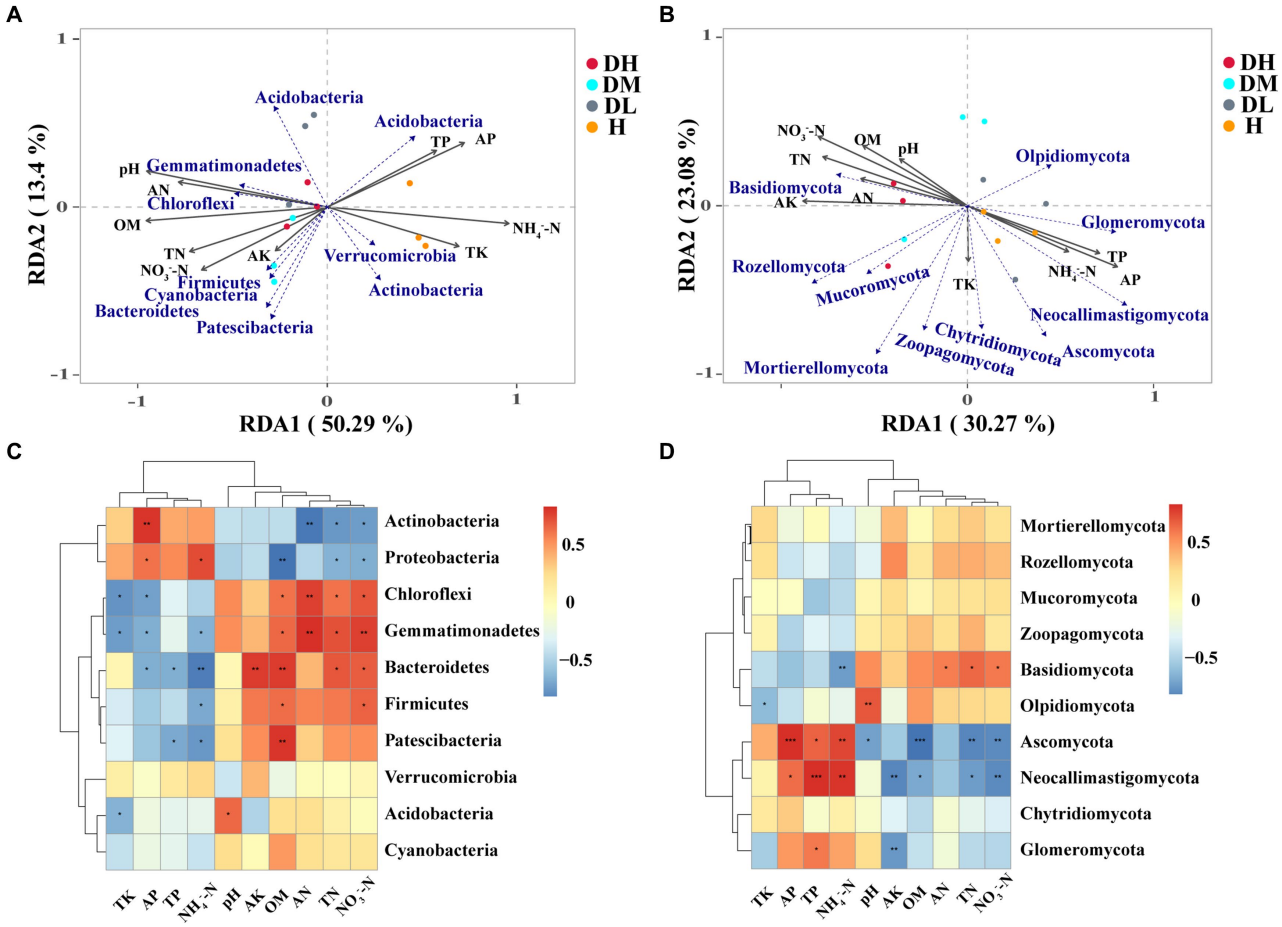
Redundancy analysis (RDA) of the relationship between soil bacterial **(A)** and fungal **(B)** community structure and soil factors under different groups. Soil chemical properties that affect the phylum (top 10) of bacteria **(C)** and fungi **(D)** based on Spearman’s correlation analysis (*0.01 < *p* ≤ 0.05, **0.001 < *p* ≤ 0.01, ***p ≤ 0.001).

RDA and Spearman analysis showed that soil physicochemical properties including organic matter (OM), pH, NH_4_^+^-N, AP, AN, TN, NO_3_^−^-N and TK were considerably linked with bacterial community composition (Figure 4A). AP and NH_4_^+^-N were positively linked with Actinobacteria and Proteobacteria and negatively correlated with Chloroflexi, Gemmatimonadetes, Bacteroidetes, and Patescibacteria. OM, TN, AN, and NO_3_^−^-N were negatively linked with Actinobacteria and Proteobacteria and positively linked with Chloroflexi, Gemmatimonadetes, Bacteroidetes, and Firmicutes (Figure 4C). Regarding fungal community composition, NO_3_^−^-N, AK, AP, TN, and TP were associated with soil (Figure 4B). Basidiomycota showed positive correlations with TN, AN, and NO_3_^−^-N and a negative correlation with NH_4_^+^-N. Ascomycota and Neocallimastigomycota showed positive correlations with TP, AP, and NH_4_^+^-N and negative correlations with pH, AK, OM, TN, and NO_3_^−^-N (Figure 4D).

### 3.6 Comparative analysis of metabolites in healthy and TRKN soils

**Figure 5:**
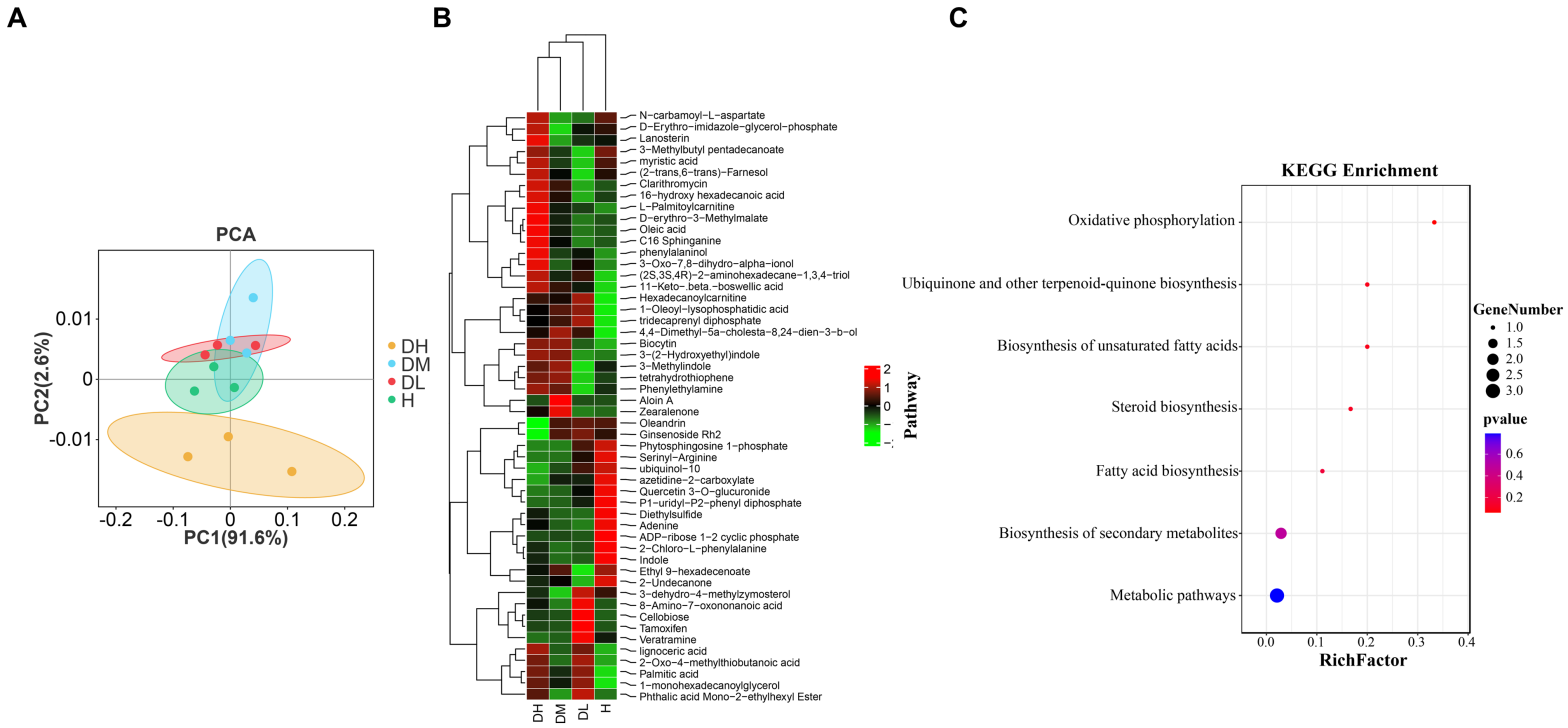
Changes in soil metabolites of tobacco plants infected by the tobacco root-knot nematode. PCA of metabolism in different treatments **(A)**. Hierarchical clustering analysis of 52 differential metabolites **(B)**. Pathway enrichment analysis of 52 differential metabolites **(C)**.

The PCA of metabolism (Figure 5A) showed that there was a significantly difference (*p* < 0.05) in the metabolic profiles between healthy and diseased soils, and that DH was clearly separated from the other three treatments. Orthogonal Partial Least Squares Discriminant Analysis (OPLS-DA) is a multivariate statistical analysis with supervised pattern recognition that can effectively screen for differential metabolites between treatments. According to the analysis of OPLS —DA model (VIP > 1, *p* < 0.05). Supplementary Figures S3A–F showed a significant separation between the control and treated groups, indicating that tobacco root knot nematode infection of the tobacco plant root resulted in significant changes in the rhizosphere soil metabolic profiles of Tobacco plants, and that these changes were also related to the degree of tobacco root knot nematode infection. Supplementary Table S3 shows that the differential metabolites in the healthy soils vs. mildly diseased soils, healthy soils vs. moderately diseased soils, healthy soils vs. severally diseased soils, mildly diseased soils vs. moderately diseased soils, mildly diseased soils vs. severally diseased soils, moderately diseased soils vs. severally diseased soils comparisons numbered 34, 22, 33, 26, 41, and 19, respectively. Across the 6 comparisons, a sum of 52 differential metabolites were observed (Supplementary Table S6). The 52 differential metabolites were subjected to hierarchical clustering analysis (HCA). Based on the expression patterns, the 52 differential metabolites could be divided into 3 clusters (Figure 5B). Twenty-seven metabolites in cluster 1 were most highly expressed in the moderately and severely diseased soils treatments, 15 metabolites in cluster 2 were most highly expressed in the healthy soils’ treatment, and 10 metabolites in cluster 3 were most highly expressed mainly in the mildly diseased soils treatment. Furthermore, in the treatment group, the differential metabolites of healthy and diseased soils were divided into two clusters, and within the diseased soil cluster into two clusters, with mildly diseased soils in a separate cluster and moderately and severely diseased soils treatments in a single cluster. The pathway enrichment of the 52 differential metabolites (Figure 5C) showed that the differential metabolites were mainly enriched in oxidative phosphorylation, ubiquinone and other terpenoid quinone biosynthesis, unsaturated fatty acid biosynthesis, steroid biosynthesis, fatty acid biosynthesis.

### 3.7 Correlation of differential microorganisms with metabolites

**Figure 6:**
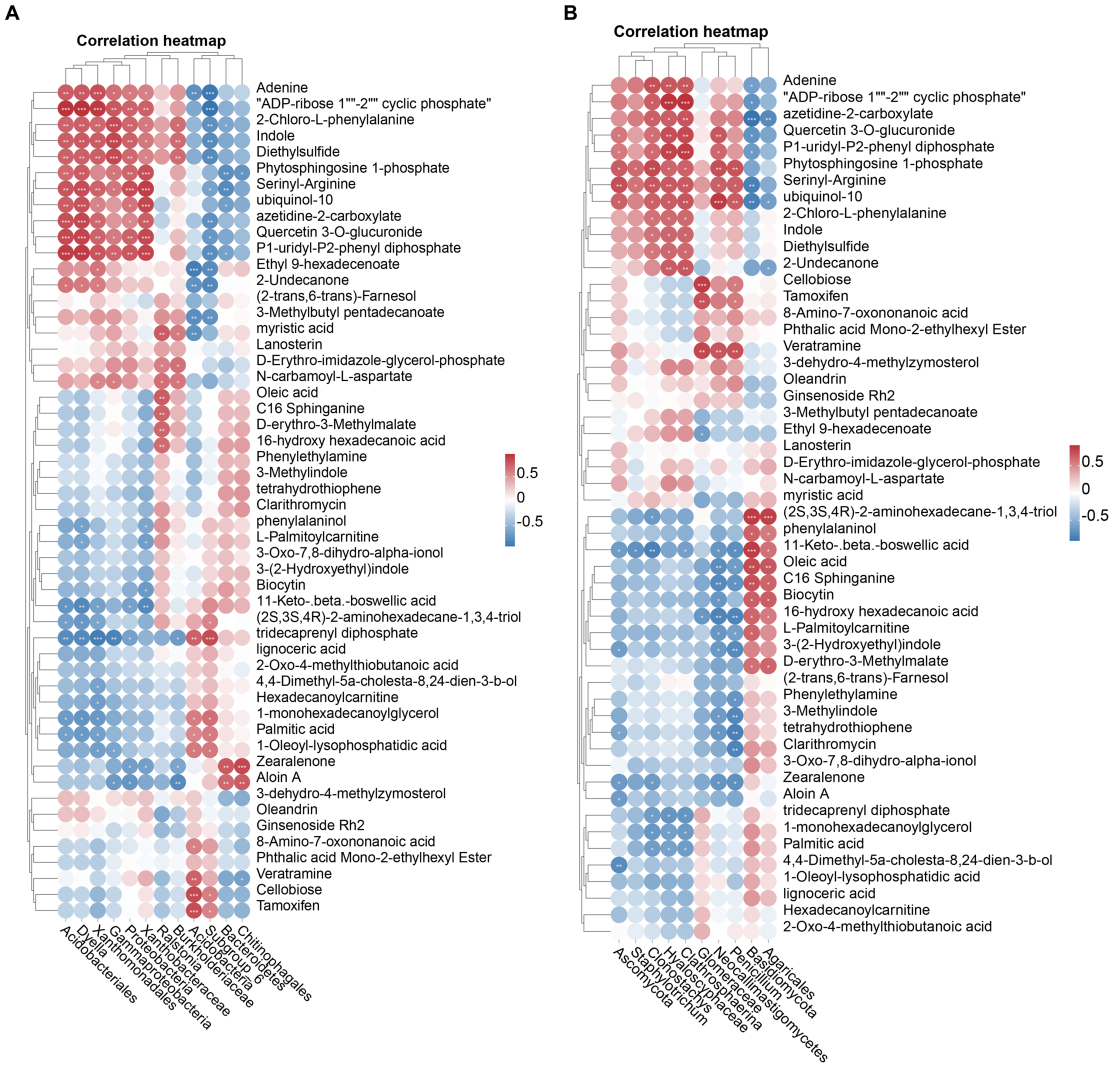
The heatmap showed a correlation between the microorganisms screened by the Linear Discriminant Analysis (LDA) effect size (LEfSe) technique and the differential metabolite. Correlation of bacterial **(A)** and fungal **(B)** with differential metabolites.

Elucidating the relationship between rhizosphere soil microorganisms and metabolites in healthy and nematode-infested tobacco plants are a necessary step towards better control of root-knot nematode invasion. Therefore, Spearman’s analyses were performed on the differential microorganisms and 52 differential metabolites. The results showed (Figure 6A) that the Proteobacteria, Gammaproteobacteria, Xanthomonadales, Acidobacteriales, Xanthobacteraceae, and *Dyella* were positively correlated with 11 metabolites, whereas they were negatively correlated with 11-Keto-beta-boswellic acid and tridecaprenyl diphosphate. The genus *Ralstonia* was positively correlated with myristic acid, D-erythro-imidazole glycerol phosphate, N-carbamoyl-L-aspartate, oleic acid, C16 sphinganine, D-erythro-3-methylmalate, 16-hydroxyhexadecanoic acid. There was also a very strong correlation between fungi and metabolites in the soil of the rhizosphere. The Hyaloscyphaceae, *Clathrosphaerina*, *Clonostachys*, were mainly positively correlated with 12 metabolites and negatively correlated with 3 metabolites. The Basidiomycota, Agaricales, were mainly positively correlated with (2S,3S,4R)-2-aminohexadecane-1,3,4-triol, phenylalaninol, 11-keto-beta-boswellic acid, oleic acid, C16 sphinganine, biocytin, 16-hydroxyhexadecanoic acid, D-erythro-3-methylmalate (Figure 6B).

## 4 Discussion

### 4.1 Microbial communities between healthy and diseased soils

Microbial communities are essential soil components that play an important role in the suppression and progression of soil-borne diseases (Garbeva et al., 2004; Yuan et al., 2020). Therefore, soil health can be evaluated by assessing its diversity and composition. Balance in the soil microbial diversity is crucial for plant health and associated with resistance to diseases in plants (Berg et al., 2017). Upon the invasion into plant roots by pathogens, the equilibrium of the soil ecosystem undergoes alteration. To foster robust crop growth, it is imperative to delve into the interplay amongst the soil milieu, plants, pathogens, and rhizosphere microorganisms. Prior research has elucidated a strong correlation between the incidence of RKN disease and the composition of soil microbial communities (Cao et al., 2022; Song et al., 2023). Therefore, using high-throughput sequencing techniques to analyze the rhizosphere soil of healthy and TRKNs infected tobacco plants. It was observed that plants infected with soil-borne diseases exhibited higher bacterial Chao1 and Shannon indices in rhizosphere soil (Zhou et al., 2019; Ji et al., 2021). Consistent with this, our study showed that the bacterial Chao1 and Shannon indexes of the moderately and mildly diseased soils were significantly higher than healthy soil. This indicates that when soil-borne pathogens attack plants, they can attract beneficial microorganisms to the rhizosphere soil by modulating root secretions (Berendsen et al., 2012). Therefore, the microbial bacterial diversity may be elevated in the infected soil when tobacco plants are infested with RKNs. However, contrasting results have also been reported, the bacterial Chao1 and Shannon indexes of the healthy soils were significantly higher than TRKNs infection soils (Huang et al., 2020). These conflicting results may be due to differences in soil type, nutrient content, climate, and disease severity.

Research has showed that soil microbial communities are essential for plant resistance against pathogens, possibly due to the interaction between specific microbial communities and plant pathogens (Sarma et al., 2015). This study showed that the significant differences in soil microbial communities between healthy and diseased soils. It may be attributed to physiological changes in the host plant after TRKNs infection. Nutrients and metabolites released by root cells infected with nematode alter the composition of root exudates, such as water-soluble carbon and metal ions, thus influencing the composition of rhizosphere microbial communities (Tian et al., 2015). Meanwhile, the abundance of rhizosphere bacteria and fungus changed significantly with different treatments. As showed in this study, the abundance of Proteobacteria, Actinobacteria, Burkholderia, *Bradyrhizobium* and *Dyella* conferred resistance to soil-borne diseases were significantly higher in healthy soils than infected soils. Proteobacteria have been reported to have the potential for to control sugar beet cyst nematodes and exhibit the highest correlation with the suppression of nematode disease (Yin et al., 2003). Actinobacteria were involved in soil nitrogen fixation, improving nutrient utilization, and promoting the production of plant growth regulators, while their metabolic products demonstrate strong antagonistic activity against plant pathogens and pests (Kaur et al., 2016; Bhatti et al., 2017). It has been showed that the higher abundance of Actinobacteria, the greater the disease resistance of the tobacco soil, probably because most of the microorganisms are highly resistant to disease (Trivedi et al., 2017). *Bradyrhizobium* exhibits excellent nitrogen fixation ability, promotes plant growth, and plays a significant role in sustainable agricultural production and ecological environment protection, as well as suppressing fungal pathogens and nematodes (Siddiqui and Shaukat, 2002). Burkholderiales can produce secondary metabolites and volatile organic compounds, which exert inhibitory effects on pathogenic bacteria. Therefore, they are crucial beneficial bacterial species in the plant rhizosphere that promote growth (Cain et al., 2000; Groenhagen et al., 2013). Furthermore, *Burkholderia-Caballeronia-Paraburkholderia* contains many beneficial environmental bacterial species that promote plant growth, bioremediation, and antibiotic activity (Beukes et al., 2017). Some species of *Dyella* can produce β-glucosidase, a bioactive component of the plant defense mechanism that improves plant resistance to pathogenic fungi (An et al., 2005; O'Kennedy et al., 2011). Ascomycota are most abundant in healthy soils and studies have showed that they have the ability to withstand harsh environments and maintain system stability (Gundlapally and Garcia-Pichel, 2006). However, the abundance of Basidiomycota, Neocallimastigomycota, Agaricales, Pseudeurotiaceae, *Chitinophaga,* and *Ralstonia* was significantly higher in diseased soils than healthy soils. The cell wall is the plant’s main barrier to pathogen invasion, and when root knot nematodes invaded the root of tobacco plants, they disrupted the cell wall, further increasing the abundance of microorganisms associated with cell wall breakdown s (Tian et al., 2015; Lu et al., 2023). These microorganisms are mainly associated with the breakdown of plant cell walls. Microorganisms involved in lignin degradation in nature belong primarily to the phylum Basidiomycota, as they can secrete the enzymes necessary for lignin degradation and are considered the main decomposers of cellulose (Kramer et al., 2016). The Neocallimastigomycota phylum plays a crucial role in the degradation of woody fibers (Wilken et al., 2021). Agaricales, Pseudeurotiaceae, *Cyathus*, and other fungi are associated with degradation of lignin and cellulose (Yamashita and Hijii, 2006; Leung et al., 2016). *Chitinophaga* is an important component in the degradation of plant cell walls (McKee and Brumer, 2015; Funnicelli et al., 2021). The structure of the plant’s cell wall skeleton was destroyed after saprophytic fungi degraded cellulose and pectin, increasing the opportunity for pathogen invasion.

In ecosystems, there are complex cooperative and competitive interactions between microorganisms in soils. Ecological networks help to detect the potential interactions among microbial species (Faust and Raes, 2012). This study found that healthy soils have higher graph density, clustering coefficient, average connectivity, and smaller average path lengths; it indicates that healthy soils have relatively complex molecular networks with close species connections, which improve the efficiency of bacterial material cycling and information transmission (Zhou et al., 2011). It suggests that bacteria in healthy soils are more sensitive, respond rapidly to external environments, and are more prone to alteration in community structure. Therefore, they can react quickly when pathogens invade (Wang et al., 2015). The results of Tax4Fun bacterial functional predictions also support these findings. Healthy tobacco rhizosphere soils have showed a greater abundance in the pathways of amino acid metabolism, carbohydrate metabolism, secondary metabolite biosynthesis, and lipid metabolism, while energy metabolism is less abundant. These results are similar to previous findings (Tong et al., 2022). Amino acids are critical components of proteins, and secondary metabolites formed by amino acids play a significant role in plant defense functions (Florencio-Ortiz et al., 2018). Amino acid metabolism converts protein breakdown into ammonia, which can inhibit nematodes in high ammonia environments (Lu et al., 2023). The biosynthesis of other secondary metabolites can antagonize several soil-borne pathogens (Han et al., 2019). Glycolysis and carbon fixation by prokaryotes are crucial sugar metabolism mechanisms and can serve as carbon sources and energy for pathogenic bacteria (Megguer et al., 2017). Moreover, Tax4Fun bacterial functional predictions indicate enhanced energy metabolism and signal transduction functions between severally diseased and moderately diseased soils bacterial communities. This result is similar to previous findings (Van Den Berg et al., 2007), it may be related to the stress response and detoxification of tobacco plants after infected by RKNs.

Compared to healthy soils and mildly diseased soils, severally and moderately diseased soils had relatively complex and closely associated molecular ecological networks, with Basidiomycota showed higher connectivity in the severally diseased soils and moderately diseased soils. Meanwhile, FUNGuild functional predictions suggest that the abundance of wood-decaying fungi in the rhizosphere soils of nematode-infected tobacco plants is higher in infected soils. Previous research has showed that most Basidiomycota fungi are primary decomposers of cell walls (Kramer et al., 2016). With the increasing severity of disease, the molecular network of fungi became more complex, which improved the relationship between Basidiomycota and other species and accelerated the dissolution of plant cell wall. This may be the reason for the progressive severity of complex infection of nematodes and other soil-borne diseases.

### 4.2 Soil physicochemical properties between healthy and diseased soils

In establishing the structure of soil microbial communities, soil physicochemical properties play a crucial role. The occurrence of plant diseases was related to soil factors that play an important role in the stability of the ecosystem. In this study, the content of TK, AP, and NH_4_^+^-N in healthy soils was significantly higher than diseased soils, the content of NH_4_^+^-N in healthy soils was 1.74 times higher than severely diseased soils. Studies have showed that the high content of NH_4_^+^-N in rhizosphere soils can reduce the invasion of plant-parasitic nematodes, as the most often implicated mechanism of killing pathogens is the transformation of ammonium into ammonia (Akhtar and Malik, 2000; Lu et al., 2023). Phosphorus and potassium can promote the development of the root system and increase the accumulation of nutrients in plants, which are beneficial to suppress soil borne diseases in crops (Huber et al., 2012). In previous study, the content of AP and TK were also identified higher in healthy soils than soil-borne diseases (Wang et al., 2023). This may explain why the contents of NH_4_^+^-N, AP and AK contents were significantly higher in healthy soils than diseased soils. On the other hand, the contents of TN, AK, AN, OM, NO_3_^−^-N, and pH are higher in diseased soils. The results of this study are consistent with the results of studies on diseased soils with higher soil pH (Wei et al., 2024). It is possible that by affecting the growth and reproduction of plant pathogenic bacteria, higher soil pH may have a direct effect on plant disease outbreaks (Shen et al., 2018). In general, the content of TN, AN, OM, AK, NO_3_^−^-N were low in soil-borne diseases soils (Wang et al., 2023). Interestingly, in this study, the TN, AN, OM, AK, NO_3_^−^-N contents of severely and moderately diseased soils were higher than other two soils. This may be the limitation of plant growth and the low rate of utilization nutrient of rate after the plant was infected by RKNs, leading to the high content of nutrients in the seriously diseases soil (Nayyar et al., 2009). Meanwhile, rotting of root tissue and necrosis caused by pathogens infection increased the deposition of root residue, thus further improving soil nutrient (Guo et al., 2021). It was also reported that the contents of AK, AN in the rhizosphere soil of the serious place Cucumber Fusarium wilt disease were higher than light and healthy place (Yang et al., 2023). Our study also found that phosphorus and ammonium nitrogen were positively correlated with beneficial flora such as Proteobacteria, Actinobacteria and Ascomycota. Therefore, the interactions between soil physicochemical properties and microbial communities have an impact on plant health.

### 4.3 Metabolites significantly healthy and diseased soils

Soil metabolites are important regulators of plant-microbe interactions, and a significant accumulation of metabolites occurs in the rhizosphere of plants following stress (Wong-Bajracharya et al., 2020). The results of the study showed that there were significant differences in metabolites between the rhizospheres of healthy and TRKN-infected tobacco plants, consistent with observations from previous studies (Wei et al., 2024). Our results showed that N-carbamoyl-L-aspartic acid, D-erythro-imidazole-glycerol-phosphate, 3-methylbutyl pentadecanoate, myristic acid, (2S,3S,4R)-2-aminohexadecane-1,3,4-triol, 11-keto-beta-boswellic acid, oleic acid, C16 sphinganine, 16-hydroxy hexadecanoic acid and D-erythro-3-methylmalate had the highest relative abundances in the severally diseased soils. At the same time, our results also indicate that oleic acid, C16 sphinganine, 16-hydroxyhexadecanoic acid, D-erythro-3-methylmalate were positively correlated with Basidiomycota, Agaricales, *Ralstonia*. Most of the compounds are organic acids and their derivatives. Organic acids, which are important secondary metabolites produced by plant metabolism and secreted by the root system into the rhizosphere soil, have been reported to be chemosensory and autotoxic to many plants, stimulating the growth of pathogenic microorganisms and exacerbating the incidence of soil-borne diseases (Kochan et al., 2019). For example, cinnamic acid can damage cucumber roots by inducing oxidative stress, which is susceptible to infection by *Fusarium oxysporum f.* sp. *cucumerinum*, promoting Fusarium wilt (Ye et al., 2004). The main autotoxin phthalic acid secreted by the roots of Lily, which promotes the development of lily wilt disease (Wu et al., 2015). Previous studies have indicated that cinnamic, myristic and fumaric acids promote colonization by *Ralstonia solanacearum* and accelerate disease progression in tobacco (Li et al., 2017). Previous studies have reported that tobacco bacterial wilt caused by *Ralstonia* is a major disease in tobacco production, often resulting in significant economic losses for farmers, while nematode infestation is frequently accompanied by a high incidence of bacterial wilt (Ahmed et al., 2022), which is consistent with the results of this study that the relative abundance of *Ralstonia* is higher in severally diseased soils. The results of previous studies have showed that organic acids, Basidiomycota, Agaricales were closely linked to the degradation of cell walls (Kramer et al., 2016; Zou et al., 2022). Therefore, the dissolution of the cell wall was accelerated with an increase in the abundance of saprophytic fungi and organic acids, which contributed to the nematode infection process during invasion and intracellular migration could be accelerated in root of tobacco plant (Danchin et al., 2010; Lu et al., 2023).

## 5 Conclusion

The physicochemical properties, microbial communities, metabolites were explored in the rhizosphere soils of tobacco plants with healthy and varying degrees of TRKNs infection. The results showed that the alpha diversity of the bacterial communities in diseased soil was higher than that in healthy soils. Microbial community composition, molecular ecological networks, and metabolic functions differed significantly between healthy and diseased soils. In addition, metabolomics results indicated that rhizosphere soil metabolites were significantly altered after tobacco plants were infected with TRKNs. The relative abundance of saprophytic fungi and organic acids was higher in severally diseased soils, providing favorable conditions for the invasion of RKNs. According to our results, to avoid serious damage to soil microbiology, we should quickly remove diseased tobacco plants from the field when the tobacco root-knot nematode infects tobacco root system. This study provides new insights into the biological control of RKNs and provides reference data on the increasing severity of RKN disease.

## Data Availability

The datasets presented in this study can be found in online repositories. The names of the repository/repositories and accession number(s) can be found here: https://ngdc.cncb.ac.cn/gsa, CRA012239 and CRA012301.

## Footnotes

^1^www.biocloud.net

^2^http://ieg4.rccc.ou.edu/MENA
