# Supplementary material for: Integration of soil microbiology and metabolomics to elucidate the mechanism of the accelerated infestation of tobacco by the root-knot nematode
Source: Front Microbiol. 2024 Aug 23;15:1455880. doi: 10.3389/fmicb.2024.1455880 (PMC11377229; doi:10.3389/fmicb.2024.1455880)
Supplement: Supplementary file 1 [file Data_Sheet_1.docx]

Supplementary Material

Rhizosphere Soil Microbial Community Assembly and Function between Healthy and Tobacco Root-Knot Nematodes Soils

Yinghua Sang^1†^, Ke Ren^2†^, Yi Chen^2^, Bin Wang^1^, Yufang Meng^3^, Wenbing Zhou^3*^, Yonglei Jiang^2*^, and Junju Xu^1*^

^1^ College of Tobacco Science, Yunnan Agricultural University, Kunming, Yunnan, China

^2^ Yunnan Academy of Tobacco Agricultural Sciences, Kunming, Yunnan, China

^3^ Yuxi Branch of Yunnan Provincial Tobacco Company, Yuxi, Yunnan, China

*** Correspondence:**Junju Xu

junjuxu007@126.com

Yonglei Jiang

jiangyatas@163.com

Wenbing Zhou

yaowufx2001@163.com

**This word file includes:**

Table S1, S3 and S5

Figure S1 to S2

**Table S1. Topological structure parameters of bacterial molecular ecological network**.

| Network Indexes | DH | DM | DL | H |
| --- | --- | --- | --- | --- |
| Total nodes | 184 | 204 | 216 | 198 |
| Total edges | 1296 | 1635 | 2589 | 2625 |
| Average degree (avgK) | 14.087 | 16.029 | 23.972 | 26.515 |
| Average clustering coefficient (avgCC) | 0.699 | 0.701 | 0.698 | 0.755 |
| Average path distance (GD) | 7.077 | 6.841 | 6.176 | 4.684 |
| Maximal degree | 23 | 27 | 45 | 43 |
| Nodes with max degree | OTU235 | OTU104; OTU1540 | OTU232 | OTU200 |
| Density (D) | 0.077 | 0.079 | 0.111 | 0.135 |
| positive correlation | 698（53.86%） | 805（49.24%） | 138（53.46%） | 1408（53.64%） |
| negative correlation | 598（46.14%） | 830（50.76%） | 1205（46.54） | 1217（46.36%） |

Note: DH represents severally diseased soil with tobacco root-knot nematode; DM represents moderately diseased soil with tobacco root-knot nematode; DL represents mildly diseased soil with tobacco root-knot nematode; H represents healthy soil

**Table S3. Topological structure parameters of fugal molecular ecological network.**

| Network Indexes | DH | DM | DL | H |
| --- | --- | --- | --- | --- |
| Total nodes | 168 | 117 | 148 | 130 |
| Total edges | 3408 | 3122 | 1460 | 1101 |
| Average degree (avgK) | 40.571 | 53.368 | 19.73 | 16.938 |
| Average clustering coefficient (avgCC) | 0.734 | 0.784 | 0.756 | 0.749 |
| Average path distance (GD) | 1.757 | 1.54 | 1.876 | 1.869 |
| Maximal degree | 161 | 114 | 146 | 129 |
| Nodes with max degree | OTU341;  OTU4576;  OTU141 | OTU4543; OTU38;  OTU41; OTU77;  OTU107; OTU65;  OTU294; OTU131 | OTU148 | OTU326 |
| Density (D) | 0.243 | 0.46 | 0.134 | 0.131 |
| positive correlation | 171（50.29%） | 1679（53.78%） | 775（53.08%） | 555（50.41%） |
| negative correlation | 169（49.71%） | 1443（46.22%） | 685（46.92%） | 546（46.36%） |

Note: DH represents severally diseased soil with tobacco root-knot nematode; DM represents moderately diseased soil with tobacco root-knot nematode; DL represents mildly diseased soil with tobacco root-knot nematode; H represents healthy soil.

**Table S5 relative abundance of functional composition of soil microbial communities under KEGG level 1 database**

| class1 | DH | DM | DL | H |
| --- | --- | --- | --- | --- |
| Cellular Processes | 0.0762a | 0.0738b | 0.0763a | 0.0748ab |
| Environmental Information Processing | 0.1091a | 0.1072ab | 0.1089a | 0.1059b |
| Genetic Information Processing | 0.0287ab | 0.0289a | 0.0280bc | 0.0275c |
| Human Diseases | 0.0354a | 0.0347ab | 0.0352a | 0.0341b |
| Metabolism | 0.7359b | 0.7403ab | 0.7364b | 0.7425a |
| Organismal Systems | 0.0146d | 0.0149c | 0.0151b | 0.0152a |

Note: DH represents severally diseased soil with tobacco root-knot nematode; DM represents moderately diseased soil with tobacco root-knot nematode; DL represents mildly diseased soil with tobacco root-knot nematode; H represents healthy soil. According to Duncan's post hoc test, different letters within the same column indicate significant differences (*p* <0.05).


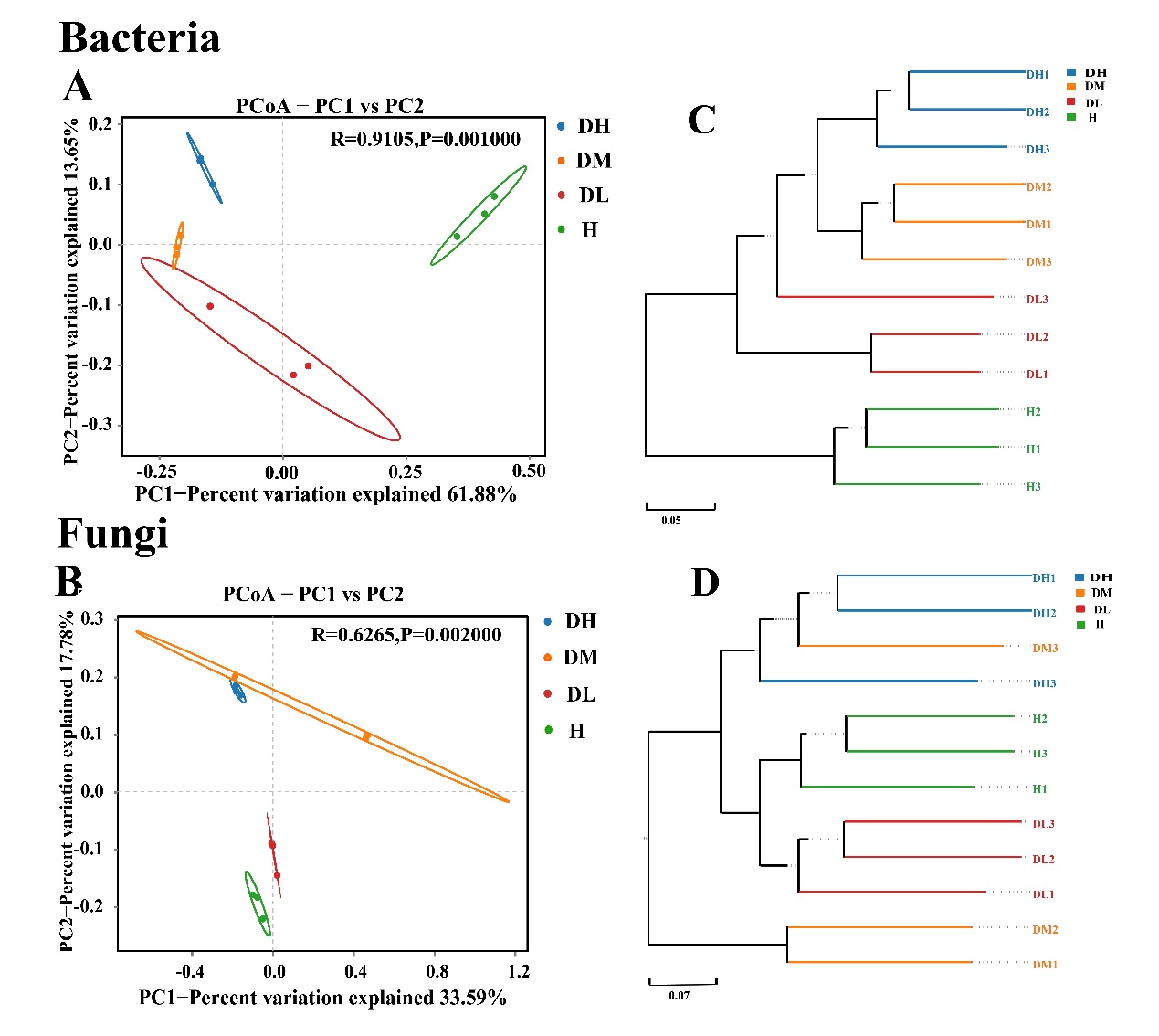


FigureS1. Beta diversity of the rhizosphere microbial community. Beta diversity of bacterial (A) and fungal (B) communities was represented using principal coordinate analysis (PCoA). Use Bray–Curtis. UPGMA clustering analysis based on Bray–Curtis for bacterial (C) and fungal (D) communities.


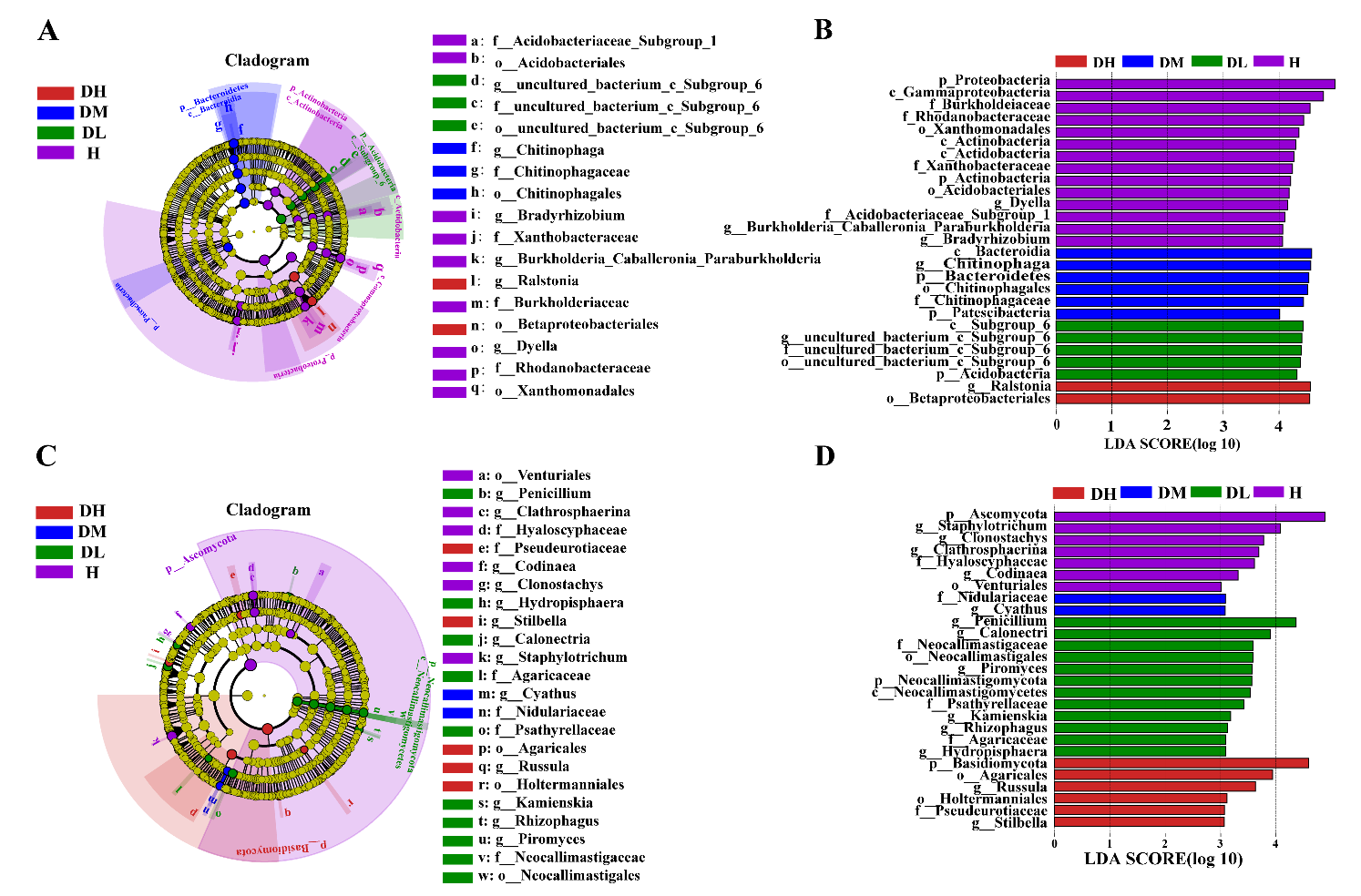


Figure S2. The linear discriminant analysis effect value (LEfSe) of bacterial and fungal. The taxonomic cladogram showed the main bacterial (A) and fungal (C) taxa in the sample community from phylum to genus (from inside to outside). The histogram showed the LDA score of bacterial (B) and fungal(D). bacterial (A) and fungal (B) communities was greater than4.0 and 3.0 when the *p* < 0.05.


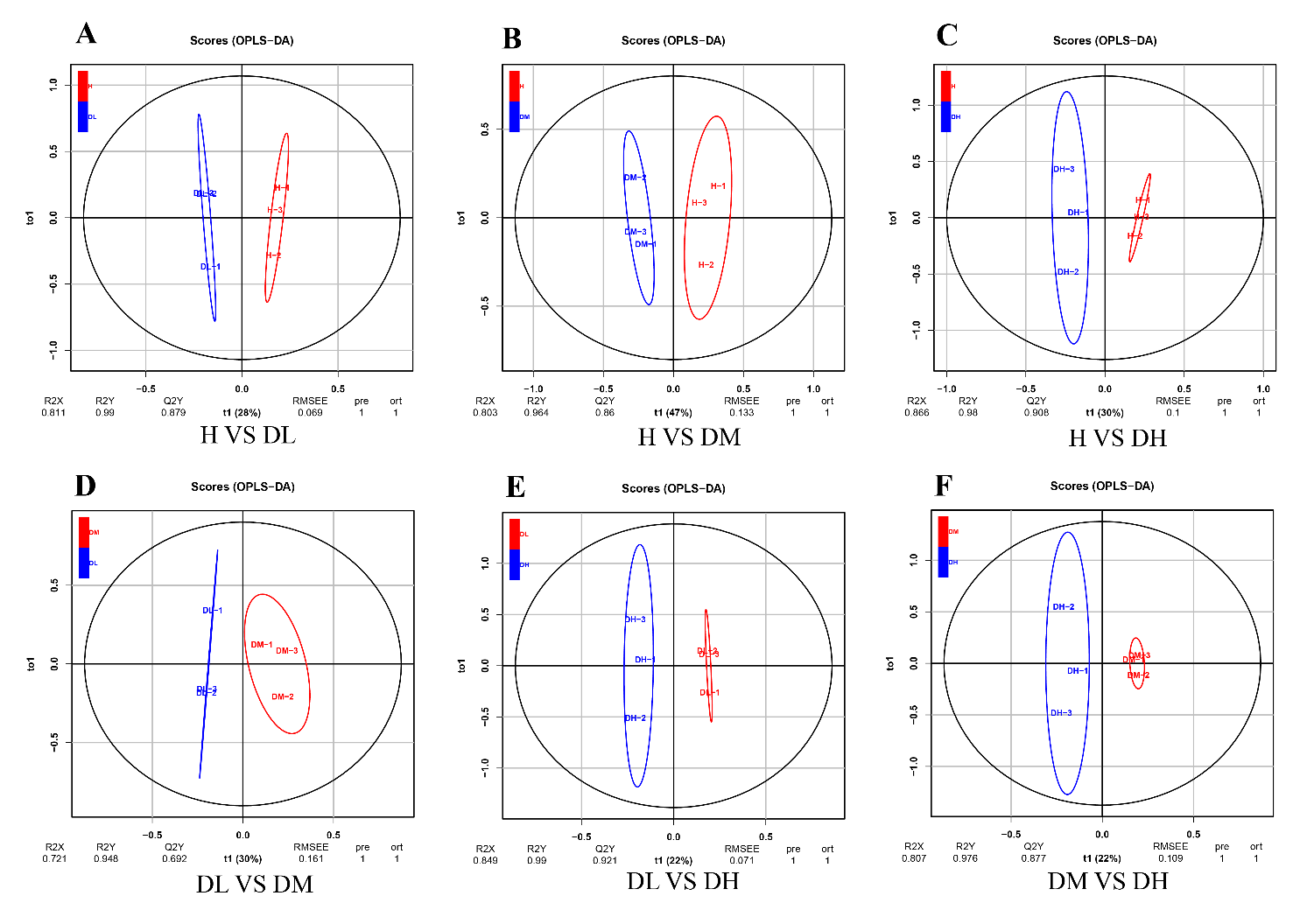


Figure S3. Plot of Orthogonal Partial Least Squares Discriminant Analysis (OPLS-DA) scores for the control and treatment groups.
